# 

**Published:** 1913-10-25

**Authors:** 


### CONTENTS.

PAGE

**Sir Almroth Wright on Woman Suffrage** ... 79

**National Military Training** ... ... ... ... 80

**Hospital and Institutional News,** including— ... 81

Nelson Hospital for Wimbledon and Merton—First Aid for Hospital Porters—The Provision of X-Ray Departments —National Insurance: Deposit Contributors—"Hospital Saturday" at Birmingham—Masonic Nursing Homes—Medical and Dental Treatment of Scholars.

**The Clinical Laboratory** ... ... ... ... 87

Alimentary Diseases.

**The International Congress** ... ... ... ... 89

Recent Surgical Developments.

**A Pioneer of Post-Graduate Teaching** ... ... 90

A Personal Appreciation of Professor R. Kutner.

**Appointments in the Tuberculosis Service** ... 91

Allegations of Corruption.

**Brief Reviews for Institutional Readers** . 92

PAGE

**Present and Future of the Poor-Law Infirmary** 93

The Resident Medical Officer's Point of View.

**The School Doctor** ... ... ... ... ... 94

The Examination of "Leavers."

**Appointments** ... ... ... ... ... 95

**The Salvation Army Mothers' Hospital** ... 96

**Buildings Contemplated** ... ... ... ... 96

**Effect of Insurance Legislation on National Character in Germany** ... ... ... 97

**The Editor's Letter-Box** ... ... ... ... 99

The Present State of Panel Medical Practice—The Threatened Strike of Asylum Nurses.

**The Non-Panel Movement** ... ... ... ... 100

**The National Medical Union Conference** ... 102

**The Institutional Worker** ... ... (Suppl.) 1

**Our Bureau of Information** ... ... „ 2

**The Book Market** ... ... ... „ 3

**Gifts and Bequests** ... ... ... „ 3

